# Real-Time Semantic Segmentation with Dual Encoder and Self-Attention Mechanism for Autonomous Driving

**DOI:** 10.3390/s21238072

**Published:** 2021-12-02

**Authors:** Yu-Bang Chang, Chieh Tsai, Chang-Hong Lin, Poki Chen

**Affiliations:** Department of Electronic and Computer Engineering, National Taiwan University of Science and Technology, Taipei City 106, Taiwan; m10802236@mail.ntust.edu.tw (Y.-B.C.); m10902119@mail.ntust.edu.tw (C.T.); poki@mail.ntust.edu.tw (P.C.)

**Keywords:** real-time semantic segmentation, deep learning, autonomous driving, image recognition, convolution neural network, edge devices

## Abstract

As the techniques of autonomous driving become increasingly valued and universal, real-time semantic segmentation has become very popular and challenging in the field of deep learning and computer vision in recent years. However, in order to apply the deep learning model to edge devices accompanying sensors on vehicles, we need to design a structure that has the best trade-off between accuracy and inference time. In previous works, several methods sacrificed accuracy to obtain a faster inference time, while others aimed to find the best accuracy under the condition of real time. Nevertheless, the accuracies of previous real-time semantic segmentation methods still have a large gap compared to general semantic segmentation methods. As a result, we propose a network architecture based on a dual encoder and a self-attention mechanism. Compared with preceding works, we achieved a 78.6% mIoU with a speed of 39.4 FPS with a 1024 × 2048 resolution on a Cityscapes test submission.

## 1. Introduction

With the rise of deep learning, self-driving cars have become increasingly popular in recent years. Semantic segmentation task is one of the fundamental and challenging tasks in computer vision and deep learning, and it can be widely applied to different applications, such as autonomous driving [1,2,3,4,5,6,7,8,9,10] and video surveillance systems [11,12], accompanying sensors on vehicles. Different from the general classification [13,14,15,16] and object detection [17,18,19] tasks, with semantic segmentation task it is more difficult to achieve good results because it needs to assign a label to each pixel in an image. However, the common classification task only needs to classify the whole image into a class. Both the classification and object detection tasks have lots of information inside the image to classify the objects.

In addition, semantic segmentation task can be differentiated into real-time semantic segmentation [6,7,8,9,10] and non-real-time semantic segmentation [1,2,3,4,5]. The goal of real-time semantic segmentation is to find the best trade-off among the accuracy, inference time, and the number of parameters in the network. On the other hand, the objective of non-real-time semantic segmentation is to achieve the highest accuracy and ignore the number of parameters and the inference time in the network.

However, most of the frameworks [1,3,5,20] in non-real-time semantic segmentation cannot be applied on edge devices due to the large model and parameters. The number of parameters of the network in non-real-time semantic segmentation is at least 10 times or even 100 times that of the number of parameters of the model in real-time semantic segmentation. Although several previous methods proposed extreme lightweight [21,22] or fast inference time [6,7,10] models for real-time semantic segmentation, the accuracy of these methods have a large gap from the accuracy of non-real-time semantic segmentation, and they may classify most of the objects incompletely.

**Figure 1:**
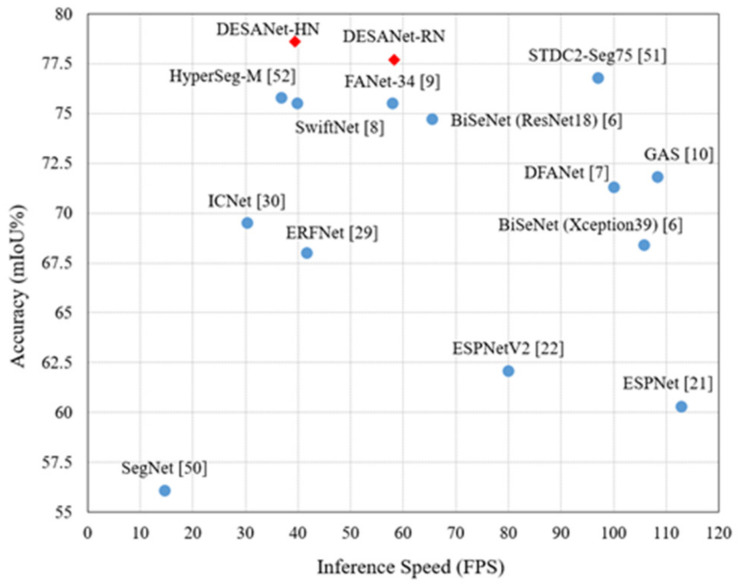
Speed–accuracy comparison using the Cityscapes Dataset. Our methods achieved a state-of-the-art trade-off between the accuracy and the inference time.

Therefore, we propose a novel and lightweight deep learning framework with a dual encoder and self-attention module that can achieve state-of-the-art results with many fewer parameters compared with other methods in the field of real-time semantic segmentation as shown in Figure 1. We trained and tested our model on the Cityscapes Dataset [23], which is based on road scenes in Germany and adjacent countries.

The main contributions are summarized as follows:

- We propose a novel and lightweight network with a dual encoder and self-attention module for real-time semantic segmentation in the field of autonomous driving;
- We designed and integrated the refinement module and the factorized atrous spatial pyramid pooling module into the network;
- We achieved state-of-the-art results in terms of accuracy with fewer parameters.

## 2. Background Knowledge

### 2.1. General Semantic Segmentation Network

In the general semantic segmentation task, the objective of the previously proposed methods is to achieve the best accuracy. Numerous works [1,2,3,4,5] were proposed to improve the performance of predicting a label for each pixel, and the results can be used in autonomous driving. Moreover, among all deep learning-based semantic segmentation approaches, the encoder–decoder structure is the most commonly adopted model, such as in [1,6,7,8,24,25,26,27]. For example, Zhao et al. [26] proposed the pyramid pooling module as the bottleneck between the encoder and decoder. It inputs the feature map to different kernel sizes, such as 1 × 1, 2 × 2, 3 × 3, and 6 × 6 convolutional layers in parallel first, and then it fuses the features of these output feature maps to extract the features from different scales.

In addition, atrous spatial pyramid pooling (ASPP) was proposed by Chen et al. [24] to extract multiscale contextual information via different approaches. They used atrous convolution with different dilation rates and global average pooling instead of the common convolution to design the ASPP [24]. The benefit of atrous convolution is that it can further enlarge the kernel size and the receptive field without increasing the number of parameters in the model. Therefore, they used large dilation rates to further extract larger feature maps, because the semantic segmentation task needs more global information from the input images. In addition, it can maintain the size of the output feature map as the same size as the input feature map, and it does not need to use an additional upsampling layer to obtain the same size. Furthermore, several other works [2,3,27] proposed the attention-based mechanism to achieve good results. Both Zhu et al.’s [2] and Huang et al.’s [3] works used the ResNet-101 [28] as the backbone in the networks. Although the preceding methods mentioned above can achieve high accuracies in the general semantic segmentation task, their number of parameters are too large to be applied on the edge devices.

### 2.2. Real-Time Semantic Segmentation Network

Due to the problem of applying the general semantic segmentation model on the edge devices mentioned in the previous section, an increasing number of real-time semantic segmentation methods have been proposed. Several previous works concentrate on reducing the inference time or the computational complexity of the model and achieve an acceptable accuracy, while others focus on improving the accuracy while still satisfying the condition of real time. For instance, Romera et al. [29] proposed the ERFNet, which uses the factorized convolution layer in the encoder of the network. The factorized convolution layer decomposes the general 3 × 3 convolution into 3 × 1 and 1 × 3 convolution layers to reduce the number of parameters in the network.

Zhao et al. [30] introduced the image cascade network, which uses three different resolutions (1024 × 2048, 512 × 1024, and 256 × 512) as the input of the three encoders and concatenates each output of the encoders as the cascade feature fusion. Moreover, they adopted the cascade label guidance strategy to guide the learning stage of low, medium, and high resolution, which is similar to deep supervision. On the other hand, the U-Net [31] structure is widely adopted in the real-time semantic segmentation task. For example, Oršic et al. [8] adopted the U-Net [31] structure to design the model and used the ResNet-18 [28] as the encoder of the network. Furthermore, they adopted spatial pyramid pooling (SPP) [26] to increase the receptive field between the encoder and decoder blocks. Moreover, Hu et al.’s [9] work also utilized the U-Net [31] structure with the ResNet-18 [28] backbone and the attention modules in each skip connections of the network for non-local context aggregation. Yu et al. [6] proposed the bilateral segmentation network (BiSeNet) which combined two encoders and one decoder. They designed the spatial path and the context path to preserve the spatial information and obtain the sufficient receptive field, respectively.

## 3. Proposed Method

In the proposed network, we designed a novel architecture from the concept of the U-Net [31] and the BiSeNet [6]. We propose a dual encoder and a single decoder with a skip connection. In this section, we introduce the overall network architecture, the proposed refinement module and the factorized atrous spatial pyramid pooling module.

### 3.1. Network Architecture

**Figure 2:**
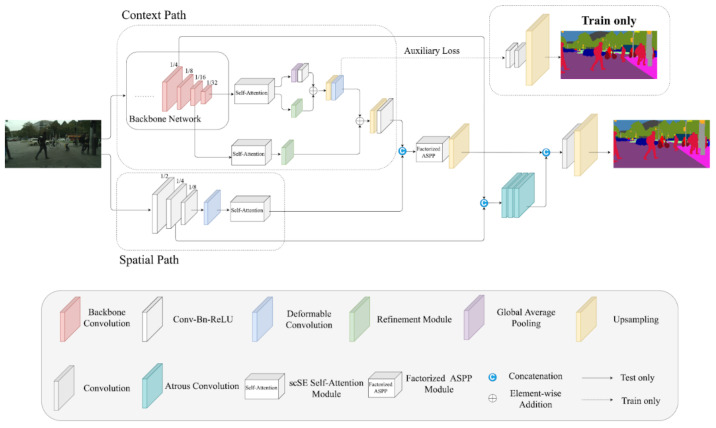
The proposed overall network architecture.

We propose a novel dual encoder–decoder architecture with skip connection for the task of semantic segmentation, which is inspired by BiSeNet [6]. Different from BiSeNet [6], we adopted the concurrent spatial and channel squeeze and excitation (scSE) [32,33] as our self-attention module and introduced a new factorized atrous spatial pyramid pooling module (FASPPM) in the model to strengthen the network’s accuracy. As shown in Figure 2, we utilized the spatial path (i.e., the bottom path) and the context path (i.e., the top path) as the dual encoder in the model, which was originally proposed by Yu et al. in [6]. The spatial path can assist the network to acquire the wealthy spatial information, because we only use three convolution layers with the stride of 2, followed by batch normalization [34] and ReLU [35] to downsample the input images. Furthermore, we used a kernel size of seven at the first convolution layer of the spatial path to let the model learn the larger receptive field. After the input images pass through three Conv-Bn-ReLU blocks, the deformable convolution [36] is used to deform the receptive field, and the receptive field is adjusted to polygon according to the different objects’ size rather than the common square size, which can boost the model’s adaptation to the transformation of the objects. The self-attention mechanism of concurrent scSE [32,33] module can extract the crucial spatial information and the channel information from the input feature maps, and it can enhance the model with a small account of additional parameters, which is helpful and efficient to introduce in the model. On the other hand, the context path can provide abundant contextual information and the sufficient receptive field. We adopted two lightweight backbone networks, namely, HarDNet-68ds [37] and ResNet-18 [28], in the context path to reduce the parameters in the overall network. In the HarDNet-68ds [37], we removed the last harmonic dense block (HDB) to further reduce the model’s size due to the fact of its large number of channels. Unlike the other backbone networks, which have a large amount of parameters, such as VGG-net [38], Inception [39], and the Densely Connected Network (DenseNet) [40], the HarDNet, designed by Chao et al. [37], not only can reduce the model’s sizes (number of parameters and the weights of the model), but it can also decrease the number of dynamic random access memory (DRAM) accesses for reading and writing the model’s parameters. It can even reduce the power consumption of edge devices that only have limited computational power. In addition, Chao et al. [37] proposed a metric called convolutional input/output (CIO) that can approximately measure the real DRAM traffic. CIO is the summation of the input tensor size and the output tensor size of all convolutions in the network. In addition to HarDNet-68ds [37], we adopted ResNet-18 [28] as the backbone network, which is the lightest version of the ResNet [28]. It is widely used in classification tasks and object detection tasks. ResNet [28] introduces the concept of residual learning into the network, which is easier to use to train a very deep neural network.

Because the semantic segmentation task generally needs to be trained for a very long time, most methods [29,41,42] adopt the pre-trained backbone on the ImageNet data set [43] to decrease the training time, and it can also assist a little bit to elevate the model’s accuracy. Moreover, both the semantic segmentation task and the classification task classify different objects with labels. Therefore, the backbone networks of our model were also pre-trained on the ImageNet data set [43] to boost the accuracy.

Different from Yu et al.’s [6] work, which only uses two encoders without any skip connection, we utilized a skip connection with three atrous convolutions [1]. The skip connection concatenates the low-level feature maps from both the context path and the spatial path. Then, the concatenated feature maps are passed through three atrous convolutions to further enlarge the receptive field with a dilation rate of 1, 3, and 6, followed according to [44], to match the high-level feature maps which have a larger receptive field. It also can enrich the detailed information from the low-level features. Moreover, we only used a skip connection to provide detailed information to the high-level features. During the experiments, we found that the accuracy would drop, and the inference time would increase dramatically when we increased the number of skip connections. In addition, if we used many skip connections in the network, the inference time of the model would be longer, and the concatenated feature maps would lead to more parameters and memory costs. On the contrary, if we did not use any skip connections, it would lead to a loss of spatial information from the low-level features. Therefore, using one skip connection is a better trade-off.

### 3.2. Refinement Module

**Figure 3:**
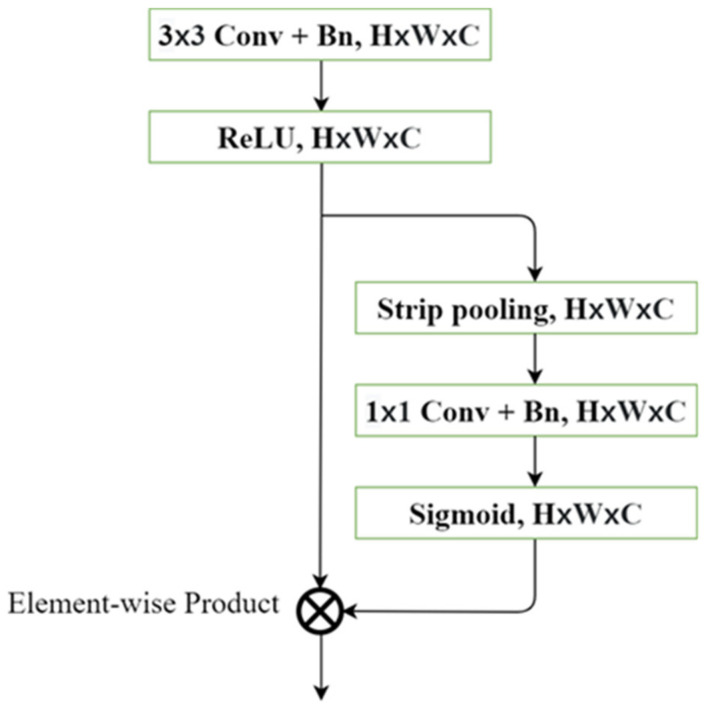
Refinement Module.

Moreover, we improved the refinement module that was initially proposed in [6]. We replaced the global average pooling with strip pooling [45] in the refinement module to capture the long-range contextual dependencies and refine the feature map as shown in Figure 3. In addition, the strip pooling [45] module can concentrate on the local details and the long-range dependencies rather than the whole information of the feature map in the global average pooling.

The strip pooling [45] averages the horizontal dimension and the vertical dimension to capture the long-range dependencies in Equation (1), where $x\in {\mathbb{R}}^{H\times W}$, $y^{h}\in {\mathbb{R}}^{H}$, and $y^{v}\in {\mathbb{R}}^{w}$. The 1D 3 × 1 convolution and 1 × 3 convolution are used along both the horizontal and vertical areas. After that, both feature maps are expanded to the size of the input feature map and fused together for each channel, followed by a 1 × 1 convolutional layer with a sigmoid function, and the input feature map is multiplied to further enhance the important information in the long-range context.

(1) $$y_{i}^{h}=\frac{1}{W}\underset{0\leq j<W}{\sum }x_{i,j},y_{j}^{v}=\frac{1}{H}\underset{0\leq i<H}{\sum }x_{i,j},y_{c,i,j}=y_{c,i}^{h}+y_{c,j}^{v}$$

### 3.3. Factorized Atrous Spatial Pyramid Pooling Module (FASPPM)

At the last stage of the encoder in the network, we propose the FASPPM to further extract the multiscale features and enlarge the receptive field, which is inspired by atrous spatial pyramid pooling (ASPP) [24]. Although ASPP [24] can boost the accuracy, it also brings a large number of parameters due to the large number of output channels due to the concatenation after the ASPP [24] and the large channel size inside the parallel atrous convolution layers [1]. Moreover, it is not suitable to embed on edge devices with limited computation.

**Figure 4:**
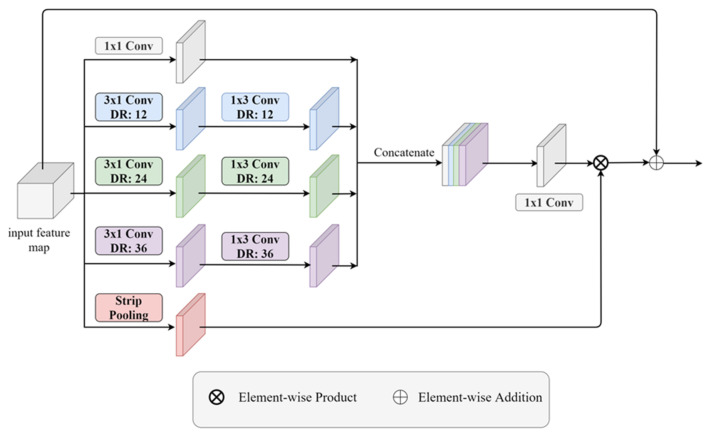
Factorized atrous spatial pyramid pooling module.

To reduce the overall number of parameters of the model, we factorized the 3 × 3 atrous convolution layer into a 3 × 1 atrous convolution layer and a 1 × 3 atrous convolution layer to decrease the computation and parameters in the model, followed by batch normalization [34] and the ReLU [35] in the ASPP, respectively, as shown in Figure 4. Furthermore, we also replaced the global average pooling with the strip pooling [45], as mentioned in the previous section, to capture the long-range dependencies and multiplied the output of the 1 × 1 convolution layer after concatenation. At last, the feature maps were added with the input feature maps as the residual learning.

### 3.4. Loss Function

We used the cross-entropy loss and the region mutual information (RMI) loss [46] as our main loss functions. In addition, we used another cross-entropy loss as the auxiliary loss to supervise the output of the context path. In the path of auxiliary loss, it included additional convolution layers and an upsampling layer, which can be ignored during the inference time. Therefore, it will not increase the number of overall parameters of the network and degrade the inference time. The total loss is shown in Equation (2), where $L_{main\_CE}$ represents the main cross-entropy loss, $L_{RMI}$ denotes the RMI loss [46], and $L_{auxiliary\_CE}$ denotes the auxiliary cross-entropy loss.

(2) $$L_{total}=L_{main\_CE}+L_{RMI}+L_{auxiliary\_CE}$$

The cross-entropy loss is commonly used in classification tasks such as semantic segmentation and object detection. The output of the decoder and the output of the auxiliary path are input to the main cross-entropy loss and the auxiliary cross-entropy loss, respectively, in Equations (3) and (4), where *N* is the number of pixels in an image, *C* is the number of classes in the training data, *y* is the ground truth label, and *p* is the estimated probability.

In addition, we adopted the online hard example mining (OHEM) [47] strategy in the cross-entropy loss to further boost the accuracy, which has been widely adopted by other methods [3,48,49]. The OHEM [47] only trains the hard example during the training, and we set the threshold to 0.7 following Reference [3]. The OHEM [47] only applied to the two cross-entropy losses:

(3) $$L_{main\_CE}=-\frac{1}{N}\overset{N}{\underset{n=1}{\sum }}\overset{C}{\underset{c=1}{\sum }}y_{n,c}log\left(p_{n,c}^{main}\right)$$

(4) $$L_{auxiliary\_CE}=-\frac{1}{N}\overset{N}{\underset{n=1}{\sum }}\overset{C}{\underset{c=1}{\sum }}y_{n,c}log\left(p_{n,c}^{auxiliary}\right)$$

In addition, because the cross-entropy loss is a pixel-wise loss, which neglects the dependencies in an image, we further used the RMI loss [46] to optimize the network. The RMI loss [46] utilizes each pixel and its neighbor pixels to denote the original pixel and maximizes the mutual information between the predicted result and the ground truth image, which can be considered as the measurement of the structural similarity. For example, the predicted probability, P, and the ground truth, Y, can be represented in Equations (5) and (6), where $p_{i}$ is in the range of $\left[0,1\right]$, $y_{i}$ is either 0 or 1, and d is the total pixels of the one pixel and the neighbor pixels, which equals to 9. Therefore, we can obtain a multi-dimensional point for each pixel in an image, which can reach high order consistency. The RMI loss [46] is shown from Equations (7) and (8), where M is a symmetric positive semidefinite matrix, $Cov\left(Y,P\right)$ is the covariance matrix of Y and P, $det\left(\cdot \right)$ is the determinant of the matrix, d is used to normalize, and C denotes the number of classes.

(5) $$P={\left[p_{1},p_{2},\ldots ,p_{d}\right]}^{T},P\in \left[0,1\right]$$

(6) $$Y={\left[y_{1},y_{2},\ldots ,y_{d}\right]}^{T},Y\in \left\{0,1\right\}$$

(7) $$M=\sum \begin{array}{c} Y-Cov\left(Y,P\right){\left(\sum P^{-1}\right)}^{T}Cov{\left(Y,P\right)}^{T}\\ M\in {\mathbb{R}}^{d\times d} \end{array}$$

(8) $$L_{RMI}=\overset{C}{\underset{c=1}{\sum }}\left(-\frac{1}{2d}log\left(det\left(M\right)\right)\right)$$

## 4. Results

In this section, we describe our training details and the data set that we used for training and testing. Then, we compare the proposed results with other previous methods in real-time semantic segmentation.

### 4.1. Training Details

We conducted all experiments using PyTorch 1.5. We utilized the stochastic gradient descent (SGD) optimizer with a momentum of 0.9, an initial learning rate of 0.005, and a weight decay of 0.0005 in training. In addition, we adopted the poly learning rate strategy [1] in which the initial learning rate was multiplied by ${\left(1-\frac{iter}{\max\_iter}\right)}^{power}$ for each iteration with the power set as 0.9. For the ResNet-18 [28] backbone and the HarDNet-68ds [37] backbone, we trained these models with the iteration set to 640,000. Both of these backbone networks were trained and tested on a single NVIDIA GTX1080Ti GPU with the batch size set as three. Moreover, we used both the fine annotation training data and the validation data to train the model to enhance the accuracy. For data augmentation, we applied a random scale between 0.5 and 2.0, random crop, random horizontal flip, random color jitter, and GridMask [50]. The random crop resolution was 1024 × 1024.

### 4.2. Benchmark and Evaluation Metrics

The Cityscapes Dataset [23] is an urban street scene data set for the semantic segmentation task that includes 2975 training images, 500 validation images, and 1525 test images in high-quality pixel-level annotations. In addition, there are 20,000 additional training images in coarse annotations. The data set was recorded in the streets from 50 different cities, which are primarily in Germany and its neighboring countries. It also covers different seasons, such as spring, summer and fall. The Cityscapes Dataset [23] comprises 19 classes: road, sidewalk, building, wall, fence, pole, traffic light, traffic sign, vegetation, terrain, sky, person, rider, car, truck, bus, train, motorcycle, and bicycle. Therefore, it is suitable for the task of autonomous driving training.

For the quantitative comparison, we needed to use a standard metric to measure the accuracy. Therefore, the standard Jaccard index, also known as the mean intersection over union (mIoU) [51], was used to compare the results, which is commonly used in the semantic segmentation task. To obtain the mIoU, we needed to upload the predicted test data on an official online server [23].

### 4.3. Comparison with State-of-the-Art Methods

A list of the quantitative comparison of the Cityscapes Dataset [23] is shown in Table 1. Our proposed method achieved a higher mIoU than the other proposed methods, and we obtained fewer parameters compared to most of the other works. For example, the results of our mIoU were higher than the method for SwiftNet [8] with many fewer parameters in the network of HarDNet-68ds [37], which is more precise and lightweight than SwiftNet [8]. Moreover, we could still achieve in real-time the semantic segmentation task even at a high resolution of 1024 × 2048. As stated in [30], a frame per second (FPS) higher than 30, in general, is sufficient for real-time semantic segmentation. The FPS is tested using TensorRT [52], which is commonly used to accelerate the inference time in real-time semantic segmentation. On the other hand, although the FPS measured in GAS [10] is higher than 100, Lin et al. [10] utilized a smaller resolution of the test data to test the FPS, and it would increase a few FPS during the inference time. Their mIoU was also much lower than the mIoU of our proposed method. Compared to other methods with the same input resolution, the proposed method using the ResNet-18 [28] backbone had the highest FPS as well.

**Figure 5:**
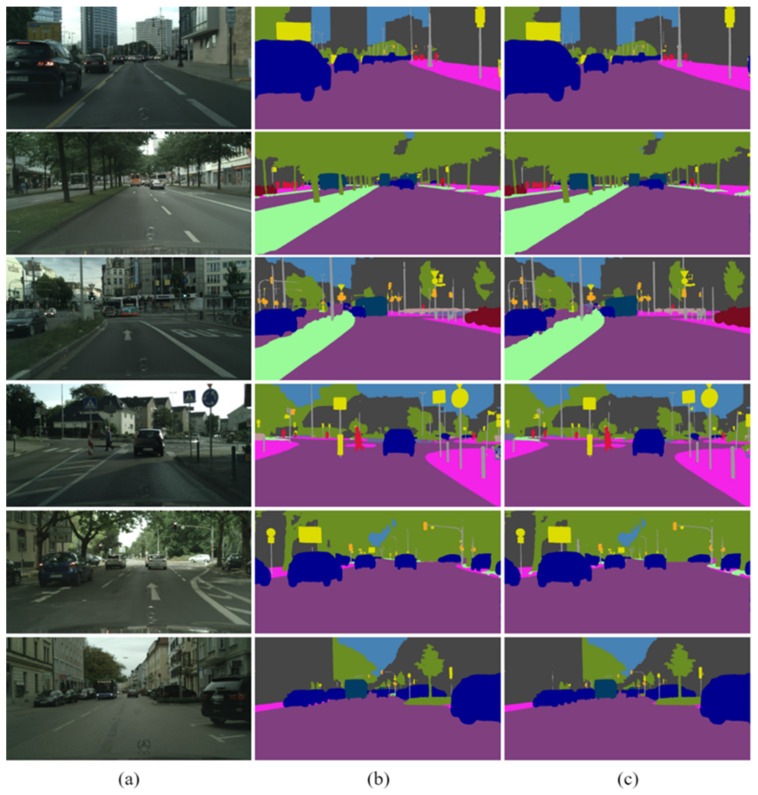
(**a**) Input image and the results of the (**b**) ResNet-18 backbone [28] and the (**c**) HarDNet-68ds [37] backbone.

**Figure 6:**
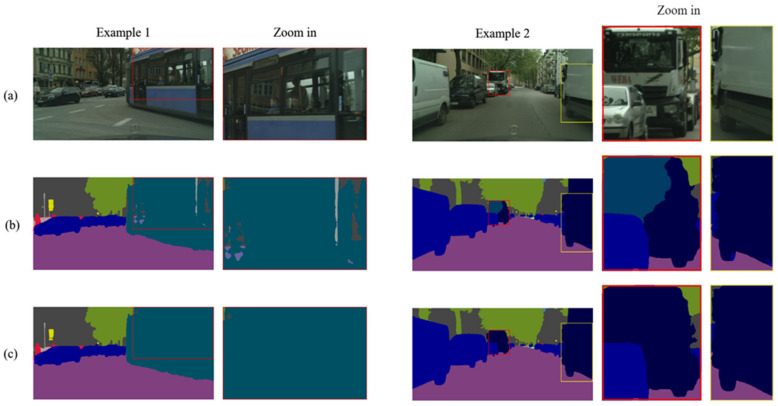
(**a**) Input image and results of the (**b**) ResNet-18 backbone [28] and the (**c**) HarDNet-68ds [37] backbone.

On the other hand, the predicted results were from six different cities of the test data set. The qualitative results of the ResNet-18 [28] backbone and the HarDNet-68ds [37] backbone are shown in Figure 5. The proposed method could classify most of the objects correctly in each pixel, such as road, sidewalk, car, bus, and vegetation. Moreover, the small objects could also be classified accurately such as traffic lights and traffic signs. Comparing the qualitative results of the network with the ResNet-18 [28] backbone and the HarDNet-68ds [37] backbone, most of the objects in the predicted results looked very similar. However, we could still obviously distinguish that the qualitative results of the HarDNet-68ds [37] backbone had a better classification with buses and trucks as shown in Figure 6.

## 5. Conclusions

In this article, we proposed a novel real-time semantic segmentation method with a dual encoder and self-attention module for autonomous driving. The dual encoder included a spatial path and a context path, which could preserve spatial information and provide abundant contextual information, respectively. The self-attention module was used to acquire crucial spatial information and channel information from the input feature maps in both the spatial path and the context path. Furthermore, we proposed the FASPPM to extract the multiscale features and enlarge the receptive field, which largely reduced the number of parameters and computational complexity than the original ASPP [24]. We also used a skip connection to provide detailed information from low-level features to the high-level features and achieved a better trade-off in accuracy, number of parameters, and FPS compared with other methods derived from the U-Net structure [31]. The proposed method achieved state-of-the-art results in terms of accuracy in the real-time semantic segmentation task with many fewer parameters using the Cityscapes Dataset [23]. For example, the results of our mIoU were higher than the SwiftNet method [8] with many fewer parameters in the network of HarDNet-68ds [37], which is more precise and lightweight than SwiftNet [8]. In addition, we could still achieve the semantic segmentation task in real time, which means the FPS was higher than 30, in general. Finally, compared with preceding works, we achieved a 78.6% mIoU with a speed of 39.4 FPS with a 1024 × 2048 resolution on a Cityscapes test submission.

## Figures and Tables

**Table 1:** Quantitative results in the test data of the Cityscapes Dataset.

Method	Resolution	GPU	mIoU	FPS	Parameters
SegNet [53]	360 × 640	Titan	56.1	14.6	29.5 M
ESPNet [21]	512 × 1024	Titan X	60.3	112.9	0.4 M
ESPNetv2 [22]	512 × 1024	Titan X	62.1	80.0	0.8 M
ERFNet [29]	512 × 1024	Titan X M	68.0	41.7	2.1 M
ICNet [30]	1024 × 2048	Titan X	69.5	30.3	26.5 M
BiSeNet (Xception39) [6]	768 × 1536	Titan XP	68.4	105.8	5.8 M
BiSeNet (ResNet-18) [6]	768 × 1536	Titan XP	74.7	65.5	49.0 M
DFANet [7]	1024 × 1024	Titan X	71.3	100.0	7.8 M
SwiftNet [8]	1024 × 2048	GTX 1080Ti	75.5	39.9	11.8 M
FANet-34 [9]	1024 × 2048	Titan X	75.5	58.0	-
GAS [10]	769 × 1537	Titan XP	71.8	108.4	-
STDC2-Seg75 [54]	768 × 1536	GTX 1080Ti	76.8	97.0	-
HyperSeg-M [55]	512 × 1024	GTX 1080Ti	75.8	36.9	10.1 M
DESANet-RN	1024 × 2048	GTX 1080Ti	77.7	58.3	15.3 M
DESANet-HN	1024 × 2048	GTX 1080Ti	78.6	39.4	6.2 M

## Data Availability

Not applicable.
